# Activation of α7 nicotinic acetylcholine receptor retards the development of endometriosis

**DOI:** 10.1186/s12958-022-00955-w

**Published:** 2022-06-04

**Authors:** Meihua Hao, Xishi Liu, Sun-Wei Guo

**Affiliations:** 1 Shanghai Obstetrics and Gynecology Hospital, Fudan University, Shanghai, 200011 China; 2 Shanghai Key Laboratory of Female Reproductive Endocrine-Related Diseases, Fudan University, Shanghai, China

**Keywords:** α7 nicotinic acetylcholine receptor, Endometriosis, Epithelial-mesenchymal transition, Fibroblast-to-myofibroblast transdifferentiation, Fibrogenesis, Smooth muscle metaplasia

## Abstract

**Background:**

Women with endometriosis have been shown to have a reduced vagal tone as compared with controls and vagotomy promoted while vagus nerve stimulation (VNS) decelerated the progression of endometriosis in mice. Extensive research also has shown that the activation of the cholinergic anti-inflammatory pathway by VNS activates α7 nicotinic acetylcholine receptor (α7nAChR), potently reducing inflammation. Yet whether α7nAChR plays any role in endometriosis is unknown. We evaluated its expression in normal endometrium, ovarian and deep endometriotic lesions, and evaluated its role in the development of endometriosis.

**Methods:**

Immunohistochemistry analyses of α7nAChR in endometriotic lesions as well as control endometrium, and quantification of tissue fibrosis by Masson trichrome staining were performed. Mouse experiments were conducted to evaluate the impact of α7nAChR activation or suppression on lesional progression and possible therapeutic effect. Finally, in vitro experiments were conducted to evaluate the effect of activation of α7nAChR on epithelial-mesenchymal transition (EMT), fibroblast-to-myofibroblast transdifferentiation (FMT), smooth muscle metaplasia (SMM) and fibrogenesis in an endometriotic epithelial cell line and primary endometriotic stromal cells derived from ovarian endometrioma tissue samples.

**Results:**

Immunostaining of α7nAChR was significantly reduced in human endometriotic epithelial cells as compared with their counterpart in normal endometrium. Lesional α7nAChR staining levels correlated negatively with lesional fibrosis and the severity of dysmenorrhea. The α7nAChR agonist significantly impeded the development of endometriotic lesions in mouse models possibly through hindrance of EMT and FMT. It also demonstrated therapeutic effects in mice with induced deep endometriosis. Treatment of endometriotic epithelial and stromal cells with an α7nAChR agonist significantly abrogated platelet-induced EMT, FMT and SMM, and suppressed cellular contractility and collagen production.

**Conclusions:**

α7nAChR is suppressed in endometriotic lesions, and its activation by pharmacological means can impede EMT, FMT, SMM, and fibrogenesis of endometriotic lesions. As such, α7nAChR can be rightfully viewed as a potential target for therapeutic invention.

**Trial registration:**

Not applicable.

**Supplementary Information:**

The online version contains supplementary material available at 10.1186/s12958-022-00955-w.

## Introduction

Featuring the presence of endometrial-like tissues outside the uterine cavity, endometriosis is an estrogen-dependent and debilitating disease, affecting 10% of women of reproductive age [1]. It negatively impacts on the quality of life of affected patients and is a major contributing cause of dysmenorrhea, chronic pelvic pain and infertility. Due to its poorly understood pathogenesis and pathophysiology, its clinical management is still challenging [1]. Although the new generation of GnRH antagonists has been proven to be effective for treating endometriosis-associated pain [2], cost appears to be a major barrier to effective management [3]. In addition, well over 60% of endometriosis patients hold a negative attitude towards hormonal drugs in general [4], thus it seems that the development of efficacious and economical non-hormonal medical treatment is still an unmet need waiting to be fulfilled [5–7].

Over the years, it has been recognized that endometriosis also is a chronic inflammatory disease [8], featuring NF-κB activation [9, 10] and increased production of pro-inflammatory cytokines and chemokines [11]. As such, anti-inflammatory therapy has attracted a great deal of attention. Unfortunately, clinical trials on anti-inflammatory therapy so far have been or presumed to be failed due to either lack of efficacy [12–14] or serious adverse events (such as AKR1C3 antagonist) [1] or both. Hence, there is still a need for effective suppression of inflammation without causing serious collateral damage.

In the last two decades, there is accumulating evidence for an intimate and intricate link between nervous and immune systems. In particular, vagus nerves regulate numerous central and peripheral processes through both afferent and efferent fibers [15], forming the cholinergic anti-inflammatory pathway (CAIP) [16–18]. The CAIP theory stipulates that, first, the ascending afferent vagal nerve fibers can convey the presence of inflammatory mediators resulting from either pathogen invasion or tissue injury to inform the central nervous system (CNS), and, second, the CNS releases, through efferent nerves, neuromediators that act on immune cells and modulate local inflammation to restore local immune homeostasis [19, 20]. In particular, anti-inflammatory reflex signaling can and has been proven to be enhanced by vagus nerve stimulation (VNS), resulting in significant reduction in cytokine production in a plethora of acute and progressive inflammatory conditions such as sepsis [21], asthma [22], rheumatoid arthritis [23], and stroke [24]. VNS also has been reported to attenuate lipopolysaccharide (LPS) induced acute lung injury by inhibiting neutrophil infiltration and neutrophil extracellular traps (NETs) formation [25].

Consistent with the CAIP theory, we have previously shown that women with endometriosis have a reduced vagal tone as compared with controls [26]. In addition, vagotomy facilitates while VNS decelerates the lesional progression in a mouse model of endometriosis [26]. Remarkably, non-invasive auricular VNS shows promising therapeutic effect, as evidenced by significantly reduced lesion weight and retarded lesional progression concomitant with improved hyperalgesia [26].

It has been known that vagal efferent fibers release acetylcholine (ACh) that can interact with α7-subunit nicotinic acetylcholine receptor (α7nAChR) expressed on tissue macrophages and other immune cells to suppress the production and secretion of pro-inflammatory cytokines such as TNFα, IL-1β, and IL-6 [17]. Indeed, the activation of the CAIP by VNS typically activates α7nAChR, potently reduces inflammation in peripheral tissues [27, 28].

In endometriosis, it is reported that α7nAChR is expressed in peritoneal fluid mononuclear cells (PFMCs) from patients with and without endometriosis [29]. Treatment with an α7nAChR agonist inhibited the gene and protein expression of IL-1β in PFMCs stimulated with LPS [29]. The activation of α7nAChR by an agonist significantly suppressed the formation of endometriotic lesions, which was reversed with an α7nAChR antagonist [29].

We have shown recently that, compared with normal endometrium, the immunoreactivity against α7nAChR is significantly reduced in both stromal and epithelial cells in adenomyotic lesions [30]. However, whether its expression is reduced in endometriosis has so far not been investigated. Nor do we know whether its modulation would have any effect on lesional development, and, if so, whether it can be a potential therapeutic target.

In this study, we hypothesized that, as in adenomyosis, α7nAChR expression is reduced in endometriotic lesions, perhaps more so in deep endometriosis (DE) as compared with ovarian endometrioma (OE). We also hypothesized that modulation of α7nAChR by pharmacological means would impact on lesional development. If this were the case, we would further hypothesize that the activation of α7nAChR by pharmacological means may have therapeutic potentials. Moreover, we hypothesized the arrest in lesional progression rendered by α7nAChR activation may be through the inhibition of epithelial-mesenchymal transition (EMT), fibroblast-to-myofibroblast transdifferentiation (FMT), smooth muscle metaplasia (SMM) and fibrogenesis—the molecular events known to occur in lesional progression [31–33]. This study was designed to test these hypotheses.

## Materials and methods

### Study participants

We recruited 17 patients with laparoscopically and histologically diagnosed OE and 14 DE patients, who visited Shanghai OB & GYN Hospital, Fudan University, over a time span from March, 2015 to March, 2017. We recruited these patients who were about to undergo laparoscopy due to OE or DE per gynecological and imaging examination. None of them had taken any anti-platelet, hormonal, oral contraceptives, or other medications at least 3 months prior to the surgery.

For controls, we recruited 18 age-matched patients who came to our hospital to undergo colposcopy or loop electrosurgical excision procedure (LEEP) treatment due to cervical intraepithelial neoplasia. None of them had any previous gynecological disorders and symptoms, or any evidence for endometriosis or adenomyosis per sonographic examination. The medical records of all recruited patients, including clinical symptoms and pathological reports, were carefully reviewed. Written informed consent was obtained from all patients. This study was approved by the ethics review board of Shanghai Obstetrics and Gynecology Hospital, Fudan University (approved on March 11, 2015, on file).

### Animals and chemicals

All experiments were performed in the in-house animal facility in accordance with the guidelines of the National Research Council’s Guide for the Care and Use of Laboratory Animals [34] and approved by the institutional experimental animals review board of Shanghai OB/GYN Hospital, Fudan University. A total of 60 female Balb/C mice, about 6–8 weeks old and about 18–21 g in bodyweight, were purchased from Shanghai LingChang Laboratory Animal Center (Shanghai, China) and used for this study. Among them, 20 were randomly selected as donors that contributed uterine fragments, and the remaining 40 were recipients.

The PNU-282987 and substance P (SP) were purchased from Sigma-Aldrich (St Louis, MO, USA), and methyllycaconitine citrate (MLA) from Abcam (Cambridge, UK). PNU-282987, MLA and SP were all dissolved in 0.9% sterile saline. As such, the control group used saline as a mock treatment.

### Induction of endometriosis

The mouse model of endometriosis was established by intraperitoneal injection of uterine fragments as we described previously [33, 35]. The inclusion of the full-thickness of uterine tissues appeared to be more advantageous in establishing endometriosis, as shown in baboons [36] and mouse [37]. Briefly, after one week of acclimatization, donor mice were injected i.m. with 150 μg/kg estradiol benzoate (Animal Medicine Factory, Hangzhou, China) twice within a week. The donor mice were sacrificed and their uteri were harvested on the day of induction. The harvested uterine tissues were rinsed twice with sterile saline and then cut into pieces by a pair of scissors, making sure that the maximal diameter of the fragment was smaller than 1 mm. Uterine fragments from one donor mouse were injected intraperitoneally to two recipient mice.

### Mouse experimental protocols

In mouse Experiment 1, we evaluated the effect of α7nAChR agonist (PNU-282987) and antagonist methyllycaconitine (MLA) on lesional development. PNU-282987 is a highly selective α7nAChR agonist and MLA is a selective and potent antagonist of α7nAChR. The doses of these drugs were determined based on the conversion of dosages used in other experiments [38, 39].

A total of 24 female Balb/C mice were divided randomly into 3 groups of equal sizes. PNU-282987 (2 mg/kg/day) and MLA (2 mg/kg/day) were administrated via Alzet osmotic pumps (model 1004, DURECT Corp, Cupertino, CA, USA) one week before the induction of endometriosis. The osmotic pump ensured consistent and controlled release of its contents for 4 weeks. For control mice, the same pumps containing an equal amount sterile saline only were administrated one week before the induction of endometriosis. For all mice, their bodyweight was recorded weekly (every 7 days) and the hotplate test was administered right before the induction procedure and then weekly until sacrifice. Three weeks after induction, all mice were sacrificed. Their abdominal cavity was immediately opened up, and all visible endometriotic lesions were carefully excised from the surrounding tissue, washed thrice in order to remove the mucosa and connective tissue. The lesion weight was measured 24 h after extraction and aspiration in order to minimize or eliminate any difference in water content, as reported previously [40]. Cystic appearance was observed in lesions [41]. The endometriotic tissue samples were then processed for quantification, immunohistochemistry (IHC) and Masson trichrome staining. We note that for the mouse model we used, the lesion weight seems to be a more reliable outcome measure than the number of lesions, since typically we observed that 2 or more lesions could be conjoined together, making the count a bit challenging.

To see whether PNU-282987 has any therapeutic potential to treat DE, we conducted mouse Experiment 2. We used an established mouse model of DE as described previously [40]. Briefly, one day before the induction of endometriosis, Alzet osmotic pumps (model 1004, DURECT Corp) were inserted in the nape of the neck in all mice to infuse substance P (100 μg/kg/day). Four weeks after the induction procedure, DE model was established. Sixteen Balb/C mice were randomly divided into two equal-sized groups, the control and the PNU-282987 groups. Mice in PNU-282987 group received PNU-282987 (2 mg/kg/day) [38] via Alzet osmotic pumps and mice in the control group only received equal amount sterile saline. The bodyweight and hotplate latency were evaluated every week. Three weeks after the PNU-282987 treatment, all mice were sacrificed and all lesions were excised and processed for quantification, IHC and Masson trichrome staining.

### Hotplate test

The hotplate test was performed with a Hot Plate Instrument (RB-200, Chengdu Techman Software Co. Ltd., Chengdu, China) to assess the extent of hyperalgesia as reported previously [42, 43]. Briefly, a metal plate of 19 cm × 19 cm in size was heated to a constant temperature of 55.0 ℃ ± 0.1 ℃. A plastic cylinder was set on the hotplate. Mice were brought into the cylinder and allowed to acclimatize for 10 min before the test. The latency to respond to thermal stimulus was defined as the time (in seconds) elapsed from the moment when the mouse was inserted into the cylinder until it began to lick its hind paws or jolted or jumped off the hotplate. Each mouse was tested only once in each session. The latency was calculated as the mean of 2 readings recorded at an interval of 24 h. The evaluator was unaware of the group identify of the mouse she was evaluating, and was practically blinded.

### Immunohistochemistry

Tissue samples were fixed in 4% paraformaldehyde (w/v) and then paraffin embedded. Serial 4-μm sections were obtained from each block. Hematoxylin and eosin staining were performed to further confirm pathological diagnosis. Immunohistochemistry analysis for α7nAChR (rabbit polyclonal antibody, 1:200, Abcam, Cambridge, UK), E-cadherin (rabbit monoclonal antibody, 1:100, Cell Signaling Technology, Boston, MA, USA), α-smooth muscle actin (α-SMA) (rabbit polyclonal antibody, 1:100, Abcam), and smooth muscle myosin heavy chain (SM-MHC) (rabbit polyclonal antibody, 1:100, Abcam) and desmin (mouse monoclonal antibody, 1:100, Abcam) was performed in subsequent slides via a polymeric/enzymatic HRP method.

Deparaffinization and dehydration procedures were performed as previously described [44]. Slides were immersed in citrate buffer and heated at 98 °C pressure cooker for 30 min, then cooled down to room temperature. The tissue was blocked in goat serum for 30 min at room temperature and then incubated at 4 °C with primary antibodies overnight. HRP conjugated secondary antibodies were applied at room temperature for 30 min the following day. Positive staining was visualized using 3, 3’-diaminobenzidine (JieHao Biological Technology, Shanghai, China) and counterstained with hematoxylin (JieHao). Images were obtained with the microscope (Olympus BX53, Olympus, Tokyo, Japan) fitted with a digital camera (Olympus DP73, Olympus). Five randomly selected images at 400 × magnification of each sample were taken to obtain a mean optical density (MOD) value and the mean density of staining intensity was acquired by Image-Pro Plus 6.0 (Media Cybernetics Inc, Bethesda, MD, USA). Staining was defined via color intensity. Briefly, a color mask was made, and then the mask was applied equally to all images. Subsequent measurement readings were obtained. Immunohistochemical parameters were assessed in the staining area, including integrated optical density (IOD), total stained area (S), and the MOD, which was defined as MOD = IOD/S. MOD was equivalent to the mean intensity of staining across all evaluated areas. To minimize any bias, the scorer was unaware of the group identify of the mouse she was evaluating, and was practically blinded.

For positive controls, human breast cancer tissues were used for E-cadherin, mouse liver tissues for α-SMA, human adenomyotic tissue samples for desmin and SM-MHC, and mouse lung tissues for α7nAChR. For negative controls, mouse endometriotic lesions were used with IgG from rabbit or mouse serum instead of primary antibodies. The representative photomicrographs for negative and positive staining controls are shown in Supplementary Figure S1.

### Masson trichrome staining

Masson trichrome staining was performed for detecting collagen and fibers in endometriotic tissue samples as previously described [45]. Briefly, tissue sections were deparaffinized in xylene and rehydrated in a gradient alcohol series, then were immersed in Bouin’s solution (saturated picric acid 75 ml, 10% (w/v) formalin solution 25 ml and acetic acid 5 ml) at 37 °C for 2 h. Sections were stained using the Masson’s Trichrome Staining kit (Baso, Wuhan, China) following the manufacturer’s instructions and used as previously reported. In Masson staining, those stained in blue were ollagen fibers, while those in red were muscle fibers. The areas of the collagen fiber layer stained in blue in proportion to the entire field of the ectopic endometrium were calculated by the Image Pro-Plus 6.0 as the percentage of fibrosis.

### Endometriotic epithelial cells and primary endometriotic stromal cells

The immortalized human endometriotic epithelial cell line (11Z), established by Professor Anna Strazinski-Powitz using SV40-antigen [46], was kindly provided by Dr. Jung-Hye Choi of Kyung Hee University, Seoul, Korea. Cells were cultured in RPMI 1640 medium (Gibco Laboratories, Life Technologies, Grand Island, NY, USA) supplemented with 5% fetal bovine serum (FBS) (Gibco Laboratories), 100 IU/mL penicillin G, 100 mg/mL streptomycin and 2.5 mg/mL Amphotericin B (Hyclone, Logan, UT, USA). Human endometriotic stromal cells (HESCs) were primarily cultured as reported in our previous work [31]. Briefly, the OE tissues were washed with DMEM/F-12 medium and minced into small pieces of about 1 mm^3^ in size. Tissues were digested with 0.2% collagenase II (Sigma-Aldrich) in a shaking bed for 1.5 h at 37 °C. The digested tissues were filtrated through a sterile 76-mm then a 37-mm filter. The filtrated cells were centrifuged and suspended in DMEM/F-12 medium supplemented with 10% FBS, 100 IU/mL penicillin, 100 mg/mL streptomycin and 2.5 mg/mL Amphotericin B, and seeded into a 25-cm^2^ cell culture flask and incubated at 37 °C in 5% CO_2_ in air. Cells with different treatments were used for quantitative real-time RT-PCR, Western blot, invasion assay, scratch test, cell immunofluorescence, cell contraction assay and collagen assay.

### RNA isolation and quantitative real-time PCR

Total RNA was isolated from 11Z cells and HESCs using TRIzol (Invitrogen, Carlsbad, CA, USA). cDNA synthesis was performed using the reverse transcription kit (Takara, Takara Bio, Inc., Otsu, Shiga, Japan). The abundance of mRNA was evaluated by real-time PCR using SYBR Premix Ex Taq (Takara). Expression values were normalized to the geometric mean of GAPDH measurements. The names of genes and their primers are listed in Table 1.

**Table 1:** List of primers used in the real-time RT-PCR analysis

Gene name Sequence		
E-cadherin	forward	5’- GCAGTTCTGCCAGAGAAACC-3’
	reverse	5’-TGGATCCAAGATGGTGATGA- 3’
α-SMA	forward	5’-CTGACAGAGGCACCACTGAA-3’
	reverse	5’-CATCTCCAGAGTCCAGCACA-3’
Vimentin	forward	5’ -TTGACAATGCGTCTCTGGCAC-3’
	reverse	5’ -CCTGGATTTCCTCTTCGTGGAG-3’
CCN2	forward	5’- GCCCTGACCCAACTATGATG-3’
	reverse	5’- CAGAGACGACTCTGCTTCTC-3’
LOX	forward	5’ -TGGTGGATCCAGATGTTTGA-3’
	reverse	5’- GTTGGTTGGGAGACTTTGGA-3’
GAPDH	forward	5’ -GTCTTCCTGGGCAAGCAGTA-3’
	reverse	5’- CTGGACAGAAACCCCACTTC-3’

### Western blot analysis

Cells were washed twice with PBS and lysed on ice with Radio-Immunoprecipitation Assay (RIPA) buffer (Thermo Fisher, Waltham, MA, USA) containing 1% protease inhibitor cocktail (Sigma). Protein concentration was determined using bicinchoninic acid (BCA) protein quantitative analysis kit (Beyotime, Shanghai, China). Proteins were loaded on a 6%-12% SDS-PAGE, and transferred to polyvinyl difluoride (PVDF) membranes (Sigma-Aldrich). The membranes were incubated at 4℃ overnight with the primary antibodies for E-cadherin (1:1000, Cell Signaling Technology), α-SMA (1:1000, Abcam), LOX (1:1000, Abcam), SM-MHC (1:1000, Abcam), desmin (1:1000, Abcam), GAPDH (1:1000, Beyotime) and β-tubulin (1:1000, Cell Signaling Technology). The membranes were incubated with HRP labeled secondary antibodies for 1 h at room temperature, and the signals were developed with enhanced chemiluminescence (ECL) reagents (Millipore, Burlington, MA, USA) and digitized on Image Quant LAS 4000 mini. Image quantification was carried out with Quantity One software (Bio-Rad, Hercules, California, USA).

### Immunofluorescence

The endometriotic epithelial cells (11Z) were seeded into 24-well plates and treated with buffer (PBS), activated platelets, PNU-282987 or both activated platelets and PNU-282987 for 3 days. HESCs were seeded into 24-well plates and co-cultured with PBS, activated platelets, PNU-282987 or both activated platelets and PNU-282987 for 3 days or 12 days, depending on the purpose of the experiment. Then cells were washed with PBS twice, fixed with 10% formalin (w/v), suspended in 0.5% Triton X-100 for 15 min, and blocked in normal goat serum, followed by incubation with the primary antibodies. For 11Z cells, E-cadherin (1:100, CST), α-SMA (1:100, Abcam), F-actin (1:100, Abcam) were used to evaluate the EMT and FMT, respectively. HESCs were incubated with α-SMA (1:100, Abcam), oxytocin receptor (OTR) (1:100, Abcam), SM-MHC (1:100, Abcam) and desmin (1:100, Abcam) overnight at 4 °C in darkness. After washing, cells were incubated at 37 °C for 1 h with Alexa Flour 488-conjugated goat anti-mouse IgG (1:200, CST) or Alexa Fluor 647 goat anti-rabbit (CST, 1:200) and then washed with PBS twice and the nuclei was stained with DAPI (Sigma-Aldrich). Images of cells were obtained by a laser scanning confocal microscope (Leica TCS SP5 Confocal Microscope, Leica, Solms, Germany). The experiments were performed in duplicate.

### Preparation of platelets

The platelets preparation procedure consists of two centrifuge steps, and performed as previously described [47]. Briefly, 20 ml of peripheral blood samples each from 20 healthy male volunteer donors, who had no known disease and had not taken any medication 3 months proior to the blood drawing, were collected using collection tubes containing 3.2% citrate solution. The platelet-rich plasma (PRP) was obtained by centrifugation at 1000 rpm for 10 min at room temperature. Supernatant was collected, and centrifuged at 3,500 rpm for 10 min. About 2 × 10^7^ platelets were collected from 1 ml of blood. The platelet pellets were suspended in RPMI 1640 or DMEM/F12 media and a total of 2 × 10^7^ platelets/ml was added into the cell-culture dishes. Platelets were activated using thrombin 0.5 U/mL (T8885, Sigma-Aldrich). Platelets were removed from cells by sterile PBS washing as reported previously [47].

### Scratch assay

The migratory ability of 11Z cells was assessed by the scratch assay as described previously [31]. Briefly, 11Z cells were cultured in 6-well plate (Corning, Tewksbury, MA, USA) and were allowed to grow to confluence. Then a 100 μL-sterile pipette tip was used to make a scratch horizontally. Serum-free cell culture medium and different solutions were added into the 6-well plate after washing with PBS thrice. Images were taken by a microscope (Olympus, Tokyo, Japan) at 0, 12 and 24 h after the scratch. The distance of each edge of 11Z cells was measured with Image Pro-Plus software 6.0 (Media Cybernetics, Inc, Bethesda, MD, USA). The assay was replicated 4 times, and the mean and standard deviation were calculated.

### Invasion assay

Biocoat 24-well Matrigel transwells were used in this assay as we reported previously [31]. Briefly, the Matrigel matrix and serum-free RPMI 1640 (Thermo Fisher, Waltham, MA, USA) culture medium were mixed in a ratio of 1:8, and then 50 μL of the mixture was added to a 24-well transwell insert and solidified in a 37 °C incubator for 30 min to form a thin gel layer. 11Z cells were detached by using 0.25% Trypsin–EDTA solution, and the cell density was adjusted to 5 × 10^5^ cells/mL. Two hundred μL of cell solution was poured into the top of the filter membrane in a transwell insert, and 600 μL of media with or without treatment was added into the basolateral side, then the cells were incubated at 37 °C in 5% CO_2_ air for 48 h. Transwell inserts were fixed by 95% alcohol, crystal violet stained, and counted under a microscope. Cells were imaged underneath an inverted microscope and counted in different fields of view to obtain an average number of cells. The invasion index was defined to be the mean counts of the infiltrated cells under × 200 magnification.

### Cell contraction assay

Cell contractility in vitro with different treatments was evaluated by cell contraction assay (Cell Biolabs, San Diego, CA, USA) according to the manufacturer’s introductions as we reported previously [48]. All solutions were kept on ice throughout the entire experiment. Three hundred forty μL of neutralization solution, 2.46 mL 5 × medium and 9.54 mL collagen solution were mixed well in a centrifuge tube to prepare for the cold collagen gel working solution. Cells were harvested and resuspended in DMEM/F12 medium at 2–5 × 10^6^ cells/mL. The collagen lattice was prepared by mixing 2 parts of cell suspension and 8 parts of cold collagen gel working solution. 24-well plates (Corning, Tewksbury, MA, USA) were coated with 0.5 mL of the cell-collagen mixture per well and incubated at 37℃ for one hour. After collagen polymerization, 1.0 mL of culture medium with different treatments was added atop each collagen gel lattice. The cultures were incubated for 72 h and collagen gels were gently released from the sides of the culture dishes. The collagen gel size change was measured with a ruler at 3, 24 and 48 h after released.

### Soluble collagen assay

Sircol soluble collagen assay (Biocolor, Carrickfergus, UK) was used to evaluate the amount of soluble collagens produced by HESCs (*n* = 7) after co-cultured with different treatments for 72 h following the manufacturer’s introductions as we previously reported [48]. The cell culture medium was collected and then centrifuged to discard the particulate materials. Low protein binding 1.5 mL conical microcentrifuge tubes (Eppendorf, Hamburg, Germany) were used to mix 1.0 mL of cell culture supernatant and 200 μL of cold Collagen Isolation and Concentration Reagent (Biocolor). DEME/F12 medium was used as blank controls and the mixture was incubated overnight at 4℃. Tubes were centrifuged at 12,000 r.p.m for 10 min without delay the next day and a micropipette was used to slowly remove 1,000 μL of supernatant from each tube. Then 1.0 mL of Sircol Dye Reagent (Biocolor) and 100 μL sample were added to each tube. The tubes were capped and placed in a gentle mechanical shaker for 30 min, and then were transferred to a microcentrifuge and spun at 12,000 r.p.m. for 10 min. The tubes were carefully inverted and drained. Seven hundred and fifty μL of ice-cold Acid-Salt Wash Reagent (Biocolor) was gently added to the collagen-dye pellet and tubes were centrifuged at 12,000 r.p.m for 10 min. The wash was drained into a waste container and the residual fluid was carefully removed from the tip of the tubes using cotton wool buds. Two hundred and fifty μL of Alkali Reagent were added to the reagent blanks, standards and samples, and after that a vortex mixer was used for thorough mixing. Then, 200 μL of each sample was transferred to individual wells of a 96 micro-well plate. The absorbance value at 555 nm was measured within 2 h by a microplate reader (Biotek, Winooski, VT, USA) and collagen concentrations were read from the standard curve.

### Statistical analysis

The comparison of distributions of continuous variables between or among 2 or more groups was made using the Wilcoxon and Kruskal test, respectively. Pearson or Spearman rank correlation coefficient was used when evaluating correlations between 2 variables when both variables were continuous or when at least 1 variable was ordinal. To see whether there is a trend for the α7nAChR staining levels or the extent of lesional fibrosis as a function of the dysmenorrhea severity, Jonckheere’s trend test was used. Multiple linear regression analysis was used to identify factors associated with the hotplate latency. To evaluate the difference in scratch assay results and in cellular contractility, linear regression analyses were performed. *P* values of less than 0.05 were considered statistically significant. All computations were made with R 4.1.2 [49] (www.r-project.org).

## Results

### Reduced α7nAChR staining in both OE and DE lesions

**Fig. 1:**
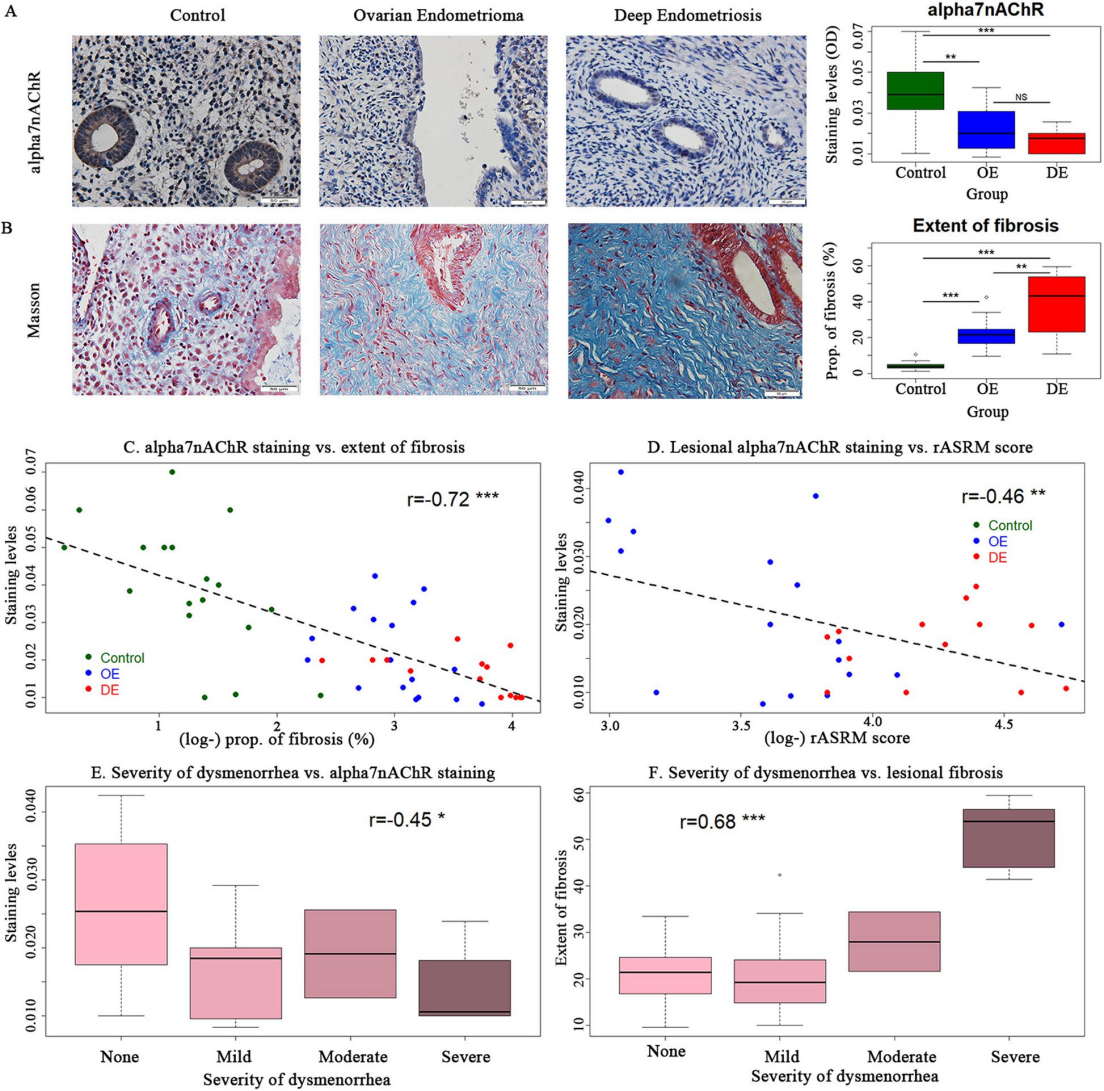
The immunohistochemistry analysis of α7nAChR in ectopic endometrium from patients with ovarian endometrioma (OE) and deep endometriosis (DE) as compared to normal endometrium from control subjects. **A** Representative immunostaining results for α7nAChR staining in normal endometrium from control subjects and ectopic endometrium from patients with OE and DE (left panel), along with boxplots summarizing the staining data (right panel). **B** Representative images of Masson trichrome staining in normal endometrium from control subjects and ectopic endometrium from patients with OE and DE (left panel), along with boxplots summarizing the staining data (right panel). Scatter plots show the relationship between α7nAChR staining levels and the extent of lesional fibrosis **C**, and between α7nAChR staining levels and rASRM scores **D**. The green, blue and red dots represent control participants, OE patients, and DE patients, respectively. The boxplots showing the relationship between α7nAChR staining levels and severity of dysmenorrhea **E**, and between severity of dysmenorrhea and the extent of lesional fibrosis **F**. Magnification = 400 × , scale bar = 50 μm. Symbols for statistical significance levels: NS: *p* > 0.05; *: *p* < 0.05; **: *p* < 0.01; ***: *p* < 0.001

We first evaluated the α7nAChR staining in normal endometrium from controls and in OE and DE lesions. The characteristics of these recruited subjects are listed in Table 2. We found a robust immunostaining of α7nAChR in normal endometrium, especially in glandular epithelium, while its staining was weaker in the stromal component (Fig. 1A). Similarly, in endometriotic lesion samples from patients with OE and DE, the α7nAChR staining was also seen mostly in the epithelial component, mainly in cell membrane (Fig. 1A). In stark contrast with the normal endometrium, however, the staining was significantly reduced in both OE and DE lesions in glandular epithelium (both *p*-values ≤ 0.003; Fig. 1A). As reported previously [50], Masson trichrome staining indicates that the extent of lesional fibrosis was significantly higher in both OE and DE lesions as compared to control endometrium (both *p*-values ≤ 4.3 × 10^–9^; Fig. 1B), especially in DE lesions. Multiple linear regression on α7nAChR staining levels incorporating age, parity, the menstrual phase at which the tissue sample was collected, and co-occurrence of uterine fibroids indicated that both OE and DE lesions were significantly associated with reduced α7nAChR staining (*p* = 0.0002 and *p* = 0.0009, respectively; *R*^*2*^ = 0.39). Multiple linear regression on the extent of tissue fibrosis incorporating age, parity, menstrual phase, co-occurrence of uterine fibroids, and α7nAChR staining indicated that α7nAChR staining levels was negatively associated with (*p* = 0.0007), while OE and DE lesions were positively associated with the extent of fibrosis (*p* = 3.0 × 10^–10^ and *p* = 2.4 × 10^–10^, respectively; *R*^*2*^ = 0.85).

**Table 2:** Characteristics of recruited patients with ovarian endometriomas, deep endometriosis and without (controls). Kruskal–Wallis test was used for age while for other data Fisher exact test was used

Variable	Control (*n* = 18)	Ovarian Endometriomas (*n* = 17)	Deep Endometriosis (*n* = 14)	*P*-value
Age (in years)				
* Mean* ± *S.D*	36.3 ± 7.0	35.6 ± 7.4	38.3 ± 5.4	0.53
* Median (Range)*	36(26–48)	35(25–48)	38(27–47	
Menstrual phase				
* Proliferative Secretory*	12 (66.7%) 6 (33.3%)	6 (35.3%) 11 (64.7%)	5 (35.7%) 9 (64.3%)	0.11
Parity				
* 0*	4 (22.2%)	6 (35.3%)	4 (28.6%)	
* 1*	13 (72.2%)	8 (47.1%)	10 (71.4%)	0.39
≥ *2*	1 (5.6%)	3 (17.6%)	0 (0.0%)	
rASRM score				
* Mean* ± *S.D*	NA	41.6 ± 21.7	74.1 ± 22.2	NA
* Median (Range)*		40 (20–112)	75 (46–114)	
rASRM stage				
* I*		0 (0.0%)	0 (0.0%)	
* II*	NA	0 (0.0%)	0 (0.0%)	NA
* III*		7 (41.2%)	0 (0.0%)	
* IV*		10 (58.8%)	14 (100%)	
Severity of dysmenorrhea				
* None*	18 (100%)	10 (58.8%)	0 (0.0%)	
* Mild*	0 (0.0%)	6 (35.3%)	4 (28.6%)	8.3 × 10^–11^
* Moderate Severe*	0 (0.0%) 0 (0.0%)	1 (5.9%) 0(0.0%)	1 (7.1%) 9 (64.3%)	
Co-occurrence with uterinefibroids				
* No Yes*	18 (100%) 0 (0.0%)	13 (76.5%) 4 (23.5%)	8 (57.1%) 6 (42.9%)	0.006
Co-occurrence with adenomyosis				
* No Yes*	18 (100%) 0 (0.0%)	16 (94.1%) 1 (5.9%)	11 (78.6%) 3 (21.4%)	0.06

Abbreviations: *NA* not applicable, *NS* not significant, *SD* Standard deviation, *Rasrm* revised American Society of Reproductive Medicine classification

*NA* not applicable

We found that the α7nAChR staining levels in eutopic and ectopic endometrium correlated negatively the extent of fibrosis (r = -0.72, p = 4.0 × 10^–9^; Fig. 1C). Lesional α7nAChR staining levels also correlated negatively the rASRM scores (r = -0.46, *p* = 0.009; Fig. 1D). In addition, the lesional α7nAChR staining levels correlated negatively with the severity of dysmenorrhea (Spearman’s r = -0.45, p = 0.012; *p* = 0.012 by Jonckheere’s trend test; Fig. 1E). The extent of lesional fibrosis correlated positively with the severity of dysmenorrhea (Spearman’s r = 0.68, *p* = 2.8 × 10^–5^; *p* = 8.8 × 10^–5^ by Jonckheere’s trend test; Fig. 1F).

### Modulation of α7nAChR affects the development of endometriosis

Given the apparent expression of α7nAChR in normal endometrium and its aberration in endometriotic lesions, we wondered whether α7nAChR would participate in the development of endometriosis. We carried out a mouse experimentation to see whether activation or inhibition of α7nAChR would affect the development of endometriosis.

We randomly divided 24 female Balb/C mice into three equal-sized groups: Control group, PNU-282987 (an α7nAChR agonist) group, and MLA group (an α7nAChR inhibitor). One week before the induction of endometriosis, osmotic pumps were inserted into these 3 groups of mice to infuse, in a controlled manner, either sterile saline, PNU-282987 or MLA for 4 weeks. Three weeks after the induction, endometriotic lesions were carefully excised and analyzed.

**Fig. 2:**
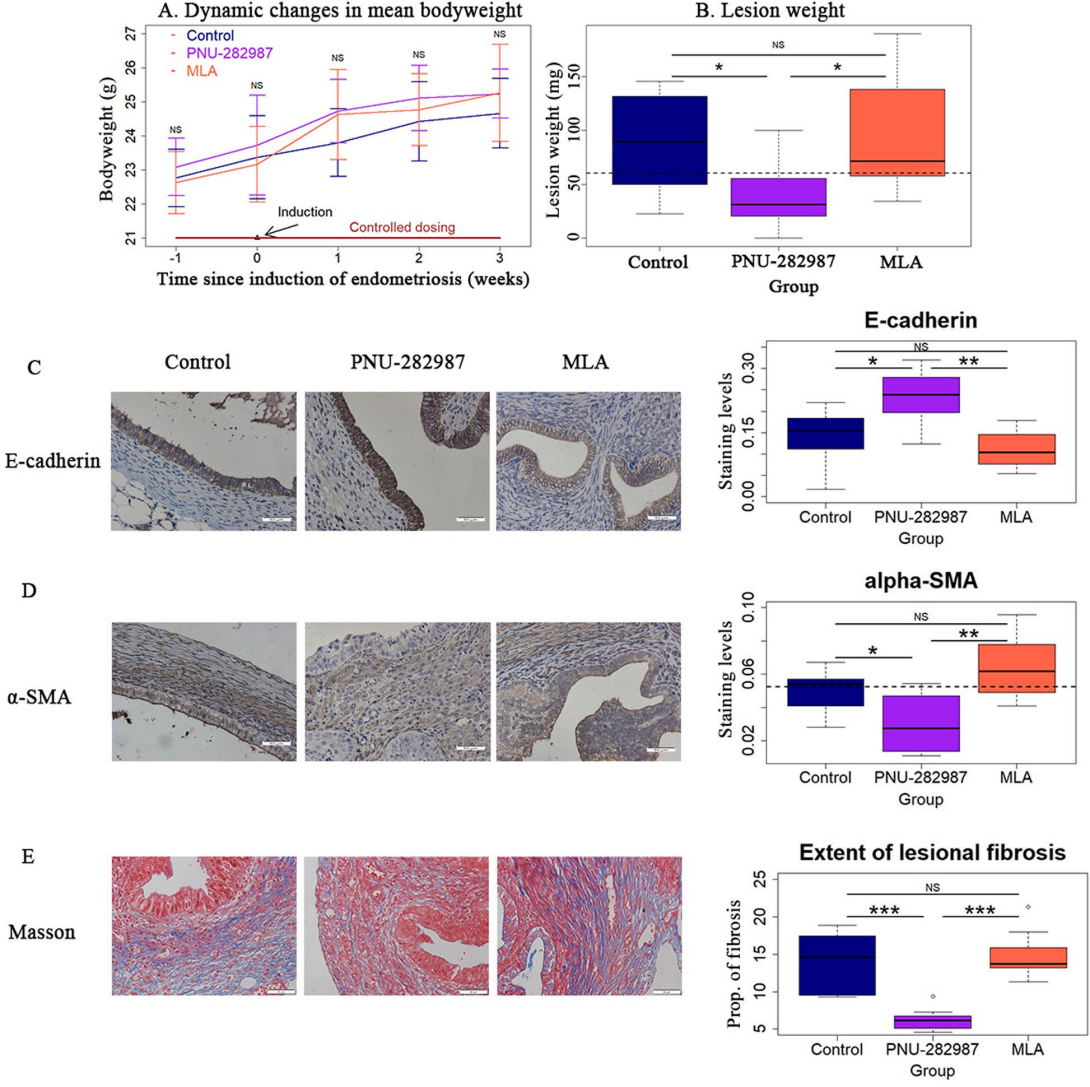
Modulation of α7nAChR affects the development of endometriosis. **A** Dynamic changes in mean bodyweight in mice from the Control, PNU-282987 and MLA groups. *n* = 8 in each group. **B** Boxplot summarizing the lesion weight among the 3 different treatment groups. Representative immunostaining results for E-cadherin **C** and α-SMA **D** in endometriotic lesions from Control, PNU-282987 and MLA mice (left panel), along the boxplots summarizing the staining data (right panel). **E** Representative images of Masson trichrome staining in endometriotic lesions in the three groups of mice (left panel), along with the boxplot summarizing the staining data (right panel). Magnification = 400 × , scale bar = 50 μm. Symbols for statistical significance levels: NS: *p* > 0.05; *: *p* < 0.05; **: *p* < 0.01; ***: *p* < 0.001

There was no significant difference in bodyweight before and after induction of endometriosis among the 3 groups of mice (all *p*-values ≥ 0.22; Fig. 2A). Endometriosis lesions were harvested and all lesions appeared to be cystic as we showed in Supplementary Figure S2. Compared with the Control group, the lesion weight in mice receiving PNU-282987 was reduced by nearly 60% (39.3 mg vs. 88.8 mg, *p* = 0.038), whereas that of mice receiving MLA was similar (*p* = 0.72; Fig. 2B). Similarly, the number of lesions in PNU-282987 mice was reduced by 46.4% (1.88 ± 0.99, vs. 3.50 ± 1.20, *p* = 0.019), whereas that of MLA mice was not significantly different from the Control mice (5.0 ± 2.0, *p* = 0.12; Supplemental Figure S3). Endometriosis was confirmed and endometriotic epithelium was visualized by H&E staining as shown in Supplementary Figure S4.

To see whether α7nAChR modulation affects EMT in endometriosis or not, we also performed IHC analysis of E-cadherin and α-SMA. Compared with control mice, the lesional E-cadherin staining levels in glandular epithelial cells from the PNU group, but not the MLA group, were significantly elevated (*p* = 0.040 and *p* = 0.28, respectively; Fig. 2C). In contrast, the staining levels of α-SMA in the glandular epithelial component in the PNU mice, but not the MLA mice, were significantly reduced as compared with the control mice (*p* = 0.049 and *p* = 0.19, respectively; Fig. 2D). Consistently, the extent of lesional fibrosis was significantly reduced in the PNU group, but not the MLA group, as compared to control mice (*p* = 0.0006 and *p* = 0.72, respectively; Fig. 2E). Taken together, these data strongly suggest that the activation of α7nAChR would partially reverse the EMT in endometriosis, hindering lesional progression.

### Treatment with an α7nAChR agonist stalls lesional progression in mouse with induced deep endometriosis

**Fig. 3:**
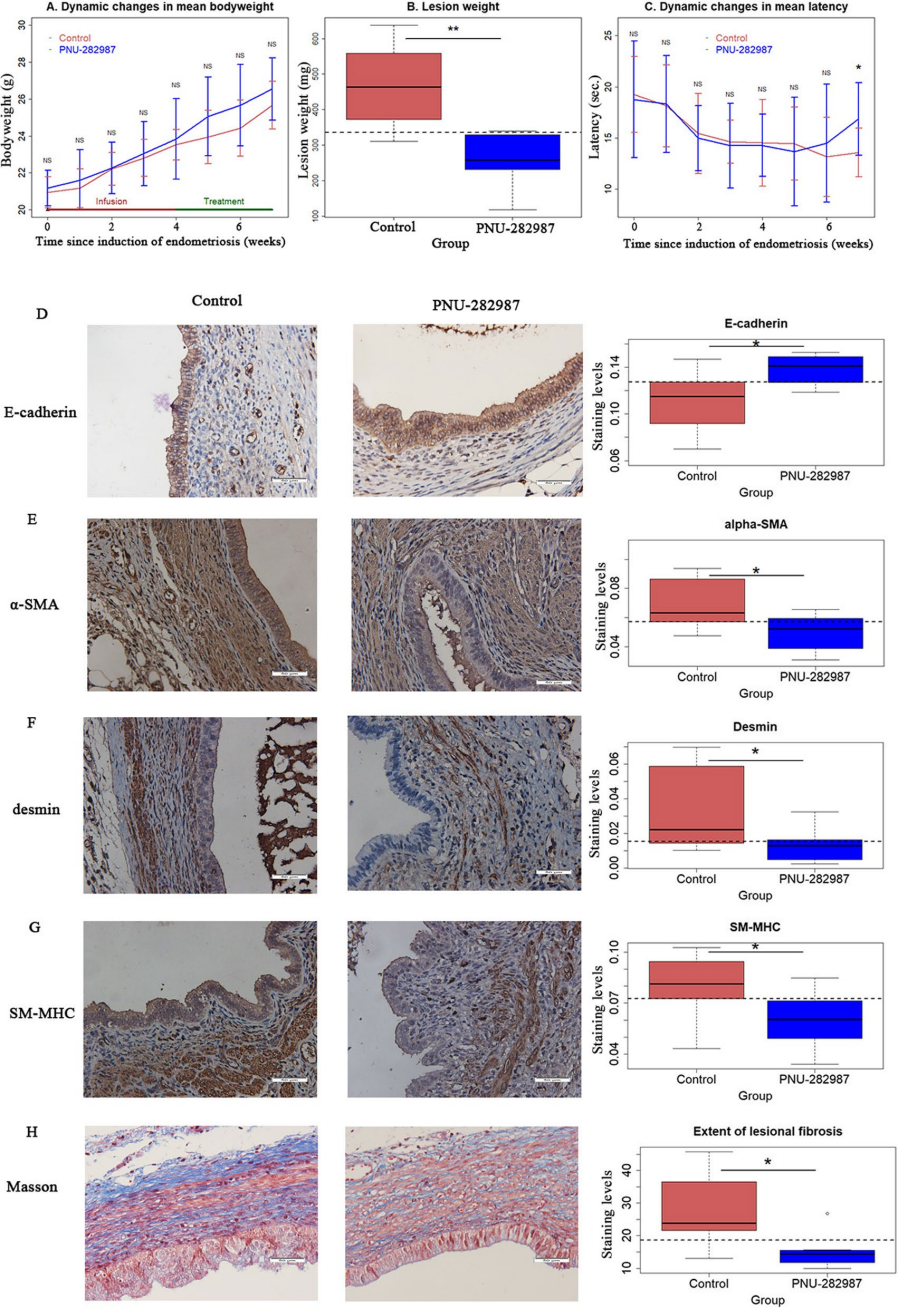
Therapeutic potentials of α7nAChR activation in treating deep endometriosis in mouse. **A** Dynamic changes in mean bodyweight in the Control and the PNU-282987 groups. **B** Boxplots of the lesion weight from the two groups of mice. **C** Dynamic changes in mean hotplate latency, tested at the indicated time points, from the two groups of mice. Representative immunostaining results for E-cadherin **D**, α-SMA **E**, desmin **F** and SM-MHC **G** in endometriotic lesions from Control and PNU-282987 treated mice (left panel), along the boxplots summarizing the staining data (right panel). **H** Representative images of Masson trichrome staining in endometriotic lesions in Control and PNU-282987 mice (left panel), along with the boxplot summarizing the staining data (right panel). Magnification = 400 × , scale bar = 50 μm. *n* = 8 in both groups. Symbols for statistical significance levels: NS: *p* > 0.05, * *p* < 0.05, ** *p* < 0.01, *** *p* < 0.001

Taking advantage of a mouse model of DE [33], we next evaluated the therapeutic potential, if any, of pharmacological activation of α7nAChR for DE. Sixteen mice were induced with DE, and 4 weeks after the induction, they were randomized to receive either PNU-282987 or saline treatment for 3 weeks. All mice survived the experiment. There was no significant difference in bodyweight before and after induction of endometriosis between the two groups (all *p*-values > 0.20; Fig. 3A).

Remarkably, compared with the control group, mice receiving PNU-282987 had their lesion weight reduced by 44.0% (261.8 ± 73.9 mg vs. 560.4 ± 115.0 mg, *p* = 0.0019; Fig. 3B), even though no difference in number of lesions between the two groups (3.63 ± 1.19 vs. 4.5 ± 1.51, *p* = 0.21; Supplemental Figure S5). There was no significant difference in hotplate latency prior to the induction of endometriosis between the two groups (all *p*-values > 0.27). When DE was induced 4 weeks after induction, there was a significant reduction in latency (*p* = 0.0013; Fig. 3C). However, the mice receiving the PNU treatment had significantly longer latency at the end of the experiment than the control mice (16.9 ± 3.6 s vs. 13.6 ± 2.4 s; *p* = 0.042; Fig. 3C).

To see whether α7nAChR agonist treatment affects EMT and fibrogenesis in mice with induced DE, we performed IHC analysis of E-cadherin, α-SMA, desmin, SM-MHC as well as Masson trichrome staining. In mice treated with PNU-282987, the lesional staining levels of E-cadherin in glandular epithelial cells were significantly elevated as compared with control mice (*p* = 0.021; Fig. 3D). In contrast, the staining levels of α-SMA, desmin and SM-MHC were all significantly decreased as compared with control mice (all 3 *p*-values ≤ 0.038; Fig. 3E-G).

Consistent with stalled EMT, FMT and SMM, we found that the extent of fibrosis was significantly reduced in mice treated with PNU-282987 as compared with controls (Fig. 3H). The extent of lesional fibrosis was negatively correlated with the epithelial staining of E-cadherin (r = -0.64, *p* = 0.007), positively with that stromal staining levels of α-SMA (r = 0.59, *p* = 0.016) and desmin (r = 0.67, *p* = 0.0045). It also correlated marginally with the lesion weight (r = 0.47, *p* = 0.066) and the latency (r = -0.46, *p* = 0.073).

Thus, after three weeks of treatment, mice with induced DE that were treated with PNU had significantly smaller lesions and longer latency as compared with untreated control mice, most likely through arresting EMT, FMT, SMM and fibrogenesis.

### Activation of α7nAChR inhibits epithelial-mesenchymal transition in endometriotic epithelial cells induced by activated platelets

**Fig. 4:**
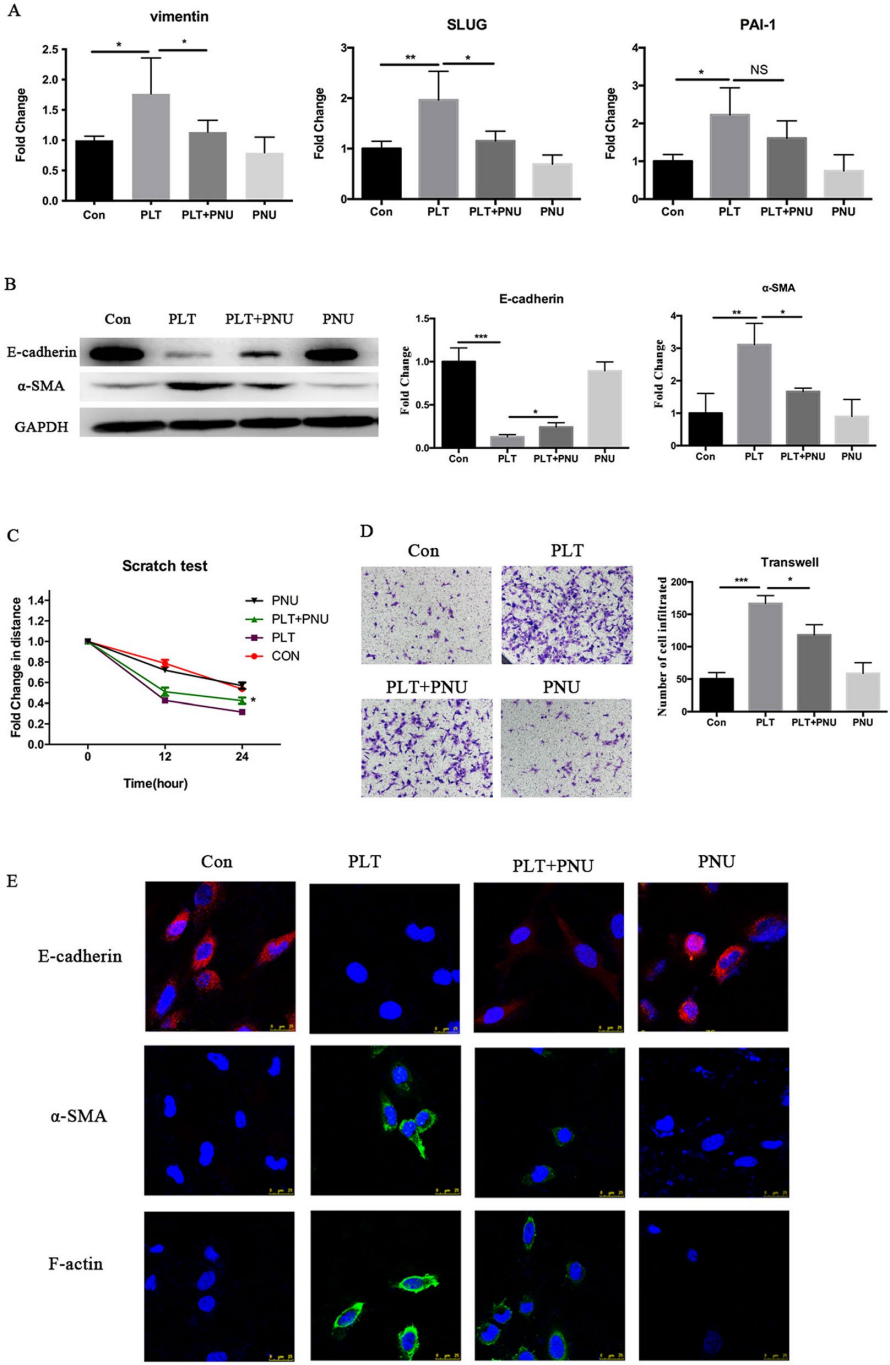
Activation of α7nAChR partially reverses platelet-induced EMT in endometriotic epithelial cells. **A** Relative fold change in gene expression levels of vimentin, SLUG and PAI-1 in 11Z cells treated with buffer, platelets activated with or without PNU-282987, or PNU-282987 alone for 48 h (*n* = 4). Values are normalized to GAPDH expression. **B** Left panel: Detection of protein levels of E-cadherin and α-SMA by immunoblotting of lysate of 11Z cells treated as in (A). Right panel: Relative fold changes of the protein levels of E-cadherin and α-SMA (*n* = 3). **C** Migratory capacity, as evaluated by the scratch assay, of 11Z cells treated with buffer, platelets with or without PNU-282987, or PNU-282987 alone for 12 and 24 h. The cells were photographed at 0 h, 12 and 24 h after scratch (*n* = 4). Fold change in migration distance was shown for different treatments as compared with controls (buffer). **D** Representative images of the invaded 11Z cells in the transwell assay under treatment indicated (Magnification: X200). Cells were added to the top of transwells coated with Matrigel and treated with buffer (Con), platelets with or without PNU-282987 (PLT, PLT + PNU), or PNU-282987 alone (PNU) for 48 h (*n* = 3). The total number of cells invaded to the bottom of transwell was then counted. Scale bar = 100 μm. **E** Representative immunofluorescence staining results of E-cadherin, α-SMA and F-actin expression in 11Z cells under different treatments as indicated. Symbols for statistical significance levels: NS: *p* > 0.05, * *p* < 0.05, ** *p* < 0.01, *** *p* < 0.001

We previously found that activated platelets stimulation could facilitate EMT in endometriotic epithelial cells [31]. This prompted us to further examine whether the activation of α7nAChR can stall EMT induced by activated platelets. We first evaluated the gene expression levels of epithelial and mesenchymal makers known to be involved in EMT, and found that vimentin, Slug and PAI-1 were all significantly upregulated in endometriotic epithelial cells co-cultured with activated platelets (all *p*-values ≤ 0.046; Fig. 4A). However, the platelet-induced upregulation of vimentin and Slug, but not PAI-1, was abrogated by PNU-282987 treatment (*p* = 0.024, *p* = 0.011 and *p* = 0.092, respectively; Fig. 4A). Consistently, Western blot analysis and immunofluorescent staining demonstrated the protein expression of E-cadherin was significantly decreased while that of α-SMA was significantly elevated in 11Z cells co-cultured with activated platelets (both *p*-values ≤ 0.0067; Fig. 4B). However, α7nAChR activation by PNU-282987 attenuated platelet-induced E-cadherin inhibition while abrogated α-SMA expression induced by platelets (both *p*-values ≤ 0.027; Fig. 4B).

We found that activation of α7nAChR attenuated the migratory propensity of 11Z cells induced by platelets (*p* = 0.043; Fig. 4C). In addition, activation of α7nAChR significantly attenuated increased invasiveness of 11Z cells induced by platelets (both *p*-values ≤ 0.016; Fig. 4D).

We also examined the expression of E-cadherin, α-SMA and F-actin through immunofluorescence after co-culturing 11Z cells with buffer, activated platelets with or without PNU-282987, or just PNU-282987 alone for 3 days. We found that the E-cadherin expression was significantly reduced while α-SMA and F-action expression were elevated when co-cultured with platelets, but PNU-282987 treatment attenuated platelet-induced suppression of E-cadherin while abrogated the expression α-SMA and F-actin induced by platelets (Fig. 4E).

Taken together, these data indicate that pharmacological activation of α7nAChR attenuated EMT induced by activated platelets in endometriotic epithelial cells.

### Activation of α7nAChR inhibits platelet-induced FMT and collagen production in endometriotic stromal cells

**Fig. 5:**
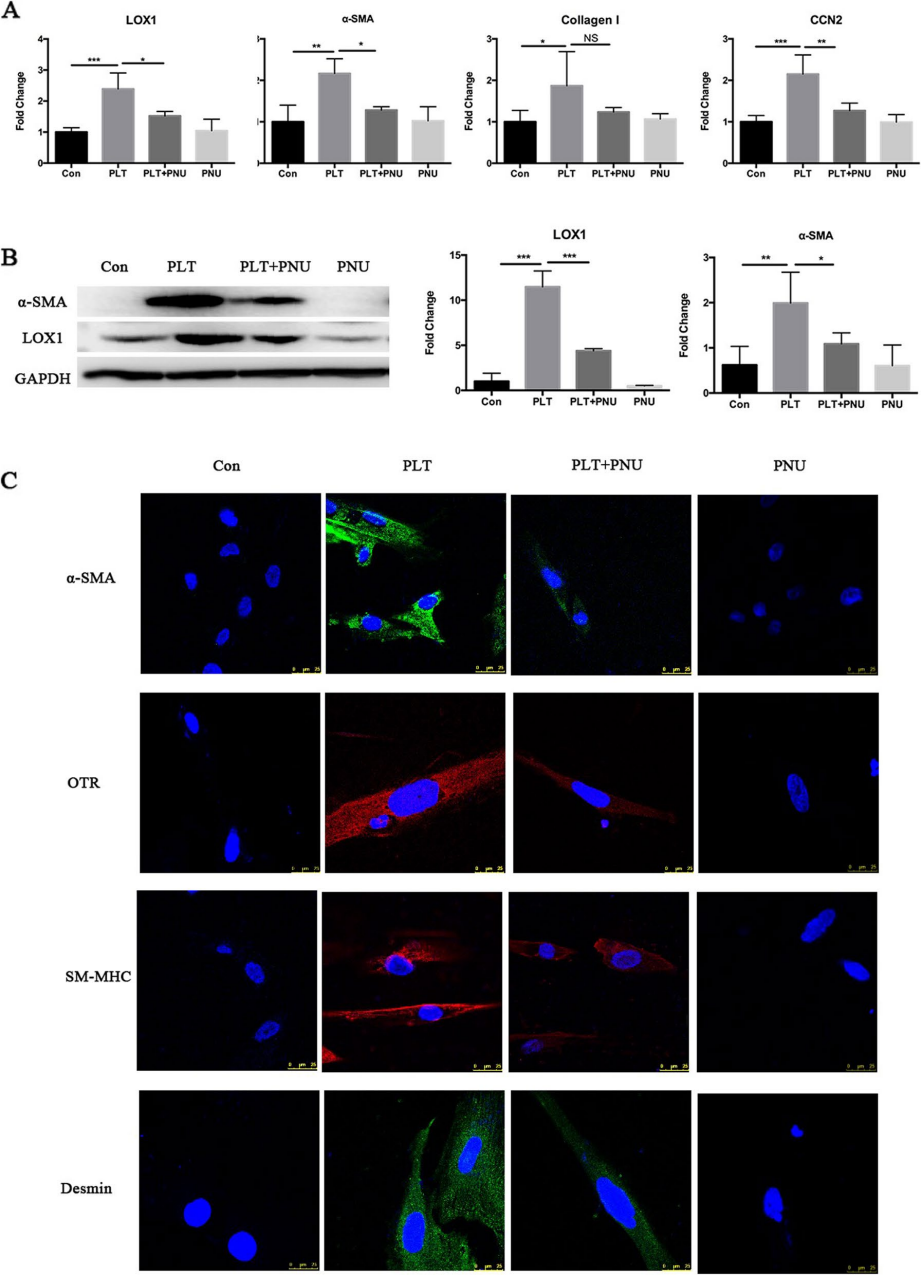
Activation of α7nAChR inhibits platelet-induced FMT in primary human endometrial stromal cells (HESCs) derived from ovarian endometrioma tissue samples. **A** Relative fold change in gene expression levels of α-SMA, LOX1, COL1A1 and CCN2 in HESCs treated with buffer (Con), activated platelets (PLT) with or without PNU-282987 (PNU), or PNU-282987 alone for 72 h (*n* = 4). **B** Left panel: Detection of protein levels of α-SMA and LOX1 by immunoblotting of lysate of HESCs treated with buffer (Con), activated platelets (PLT) with or without PNU-282987 (PNU), or PNU-282987 alone for 72 h. Right panel: Relative fold change of the protein levels of α-SMA and LOX1 (*n* = 5). **C** Representative immunofluorescence of SM-MHC, desmin, α-SMA and OTR expression in HESCs cells after indicated treatment for Day 12. Symbols for statistical significance levels: NS: *p* > 0.05, * *p* < 0.05, ** *p* < 0.01, *** *p* < 0.001

To investigate whether activation of α7nAChR impacts on platelet-induced FMT and collagen production in endometriotic stromal cells, we co-cultured HESCs with buffer, activated platelets with or without PNU-282987, or PNU-282987 alone for 72 h. We evaluated the expression levels of genes known to be involved in FMT. As expected, the gene expression levels of LOX, CCN2, α-SMA and COL1A1 were significantly elevated in HESCs co-cultured with activated platelets (all *p*-values ≤ 0.012; Fig. 5A) [31]. However, treatment with PNU-282987 significantly abrogated the expression levels of LOX, CCN2 and α-SMA (all 3 *p*-values ≤ 0.02; Fig. 5A), but not COL1A1 (*p* = 0.15; Fig. 5A). This result was corroborated by Western blot analysis for LOX and α-SMA. The protein expression of LOX and α-SMA were increased significantly (both *p*-values ≤ 0.0018; Fig. 5B), but the α7nAChR activation by PNU-282987 abrogated the LOX and α-SMA expression induced by platelets (both *p*-values ≤ 0.048; Fig. 5B).

**Fig. 6:**
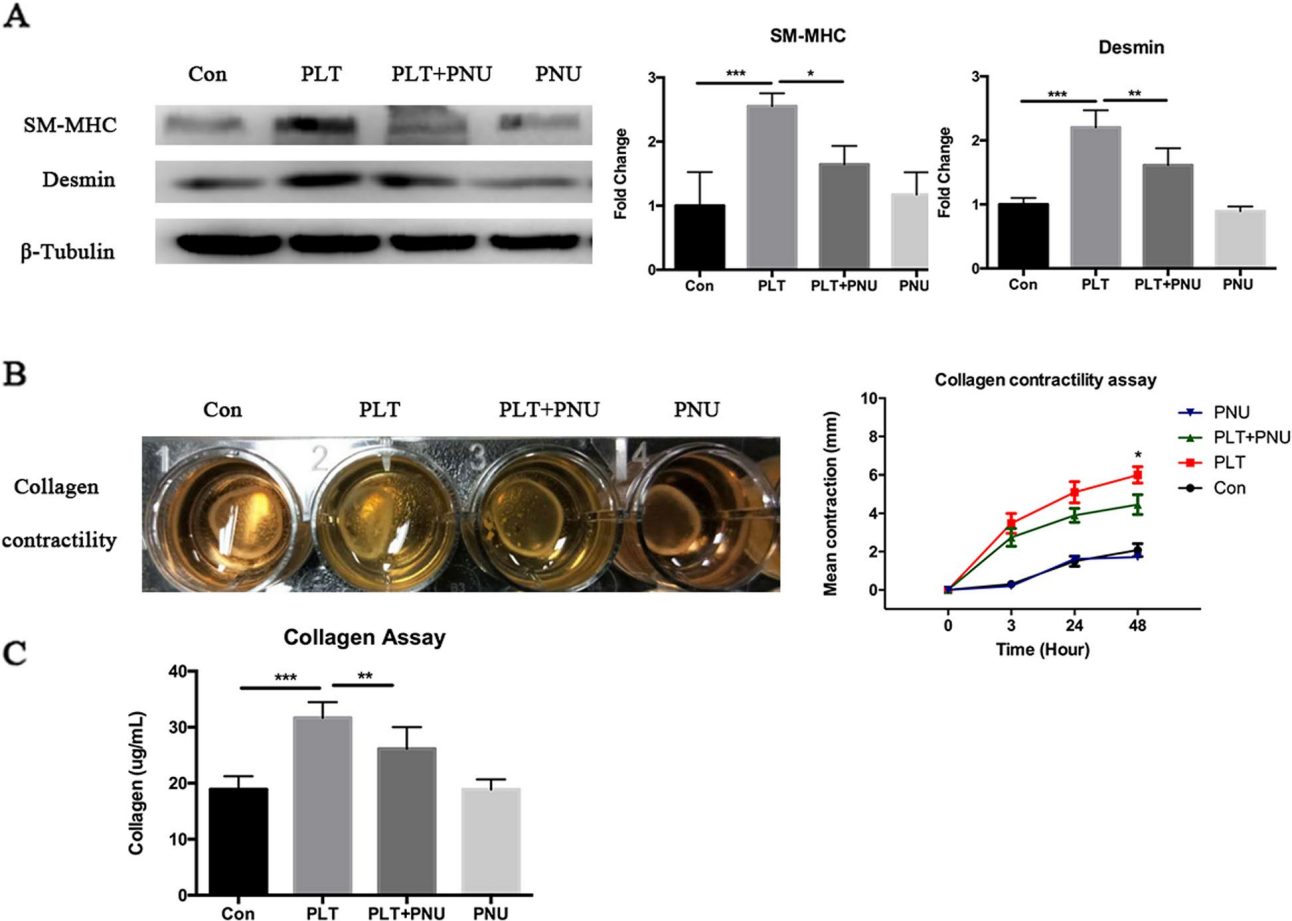
Activation of α7nAChR partially reverses platelet-induced FMT in HESCs. **A** Left panel: Detection of protein levels of SM-MHC and desmin by immunoblotting of lysate of HESCs cells treated with buffer (Con), activated platelets (PLT) with or without PNU-282987 (PNU), or PNU-282987 alone for 12 days (*n* = 4). Right panel: Relative fold change in the protein levels of SM-MHC and desmin. **B** Summary contractility results, in terms of diameter of the gel surface, for HESCs measured at 0, 3, 24 and 48 h after indicated treatment (*n* = 5). **C** The absorbance value at 570 nm (optical density, or OD) reflects the amounts of soluble collagens in the culture medium from cells treated as indicated (*n* = 7). Symbols for statistical significance levels: NS*: p* > 0.05, * *p* < 0.05, ** *p* < 0.01, *** *p* < 0.001

In addition, we found that HESCs co-cultured with activated platelets for 12 days displayed increased staining of α-SMA, OTR, SM-MHC and desmin, but this increase was abrogated by α7nAChR activation (Fig. 5C). Consistently, the protein expression level of SM-MHC and desmin were significantly elevated in HESCs co-cultured with activated platelets (both *p*-values ≤ 3.0 × 10^–4^; Fig. 6A), but this overexpression was significantly abrogated by activation of α7nAChR by PNU-282987 (both *p*-values ≤ 0.023; Fig. 6A).

Consistent with the above findings, we found that the contractility and the collagen production were significantly increased in HESCs after co-cultured with activated platelets for 72 h [31] but both the contractility and the collagen production were significantly attenuated by α7nAChR activation (both *p*-values ≤ 0.049; Fig. 6B-C).

Taken together, these data strongly indicate that activation of α7nAChR inhibits platelet-induced FMT, SMM and collagen production in endometriotic stromal cells.

## Discussion

In this study, we have shown that, similar to adenomyosis [30], the α7nAChR immunostaining is significantly reduced in endometriotic lesions, especially in DE lesions. The staining levels of α7nAChR in lesional glandular epithelium are negatively correlated with the extent of lesional fibrosis and the severity of dysmenorrhea. The α7nAChR agonist significantly impeded the development of endometriotic lesions in mouse, likely through hindrance of EMT and FMT. In mice with induced DE, treatment with an α7nAChR agonist significantly reduced the lesion weight and improved the pain behavior, which were accompanied by the arrest of EMT, FMT, SMM and fibrogenesis. Treatment of endometriotic epithelial cells with an α7nAChR agonist significantly abrogated platelet-induced EMT and invasiveness. Treatment of endometriotic stromal cells with an α7nAChR agonist also significantly attenuated platelet-induced FMT and SMM, and suppressed cellular contractility and collagen production. Taken together, these results indicate that α7nAChR is suppressed in endometriotic lesions, and its induction by pharmacological means can impede EMT, FMT, SMM, and fibrogenesis of endometriotic lesions. As such, α7nAChR can be rightfully viewed as a potential target for therapeutic invention.

Our finding of reduced lesional α7nAChR staining is consistent with our previous report of depressed vagal tone in women with endometriosis [26]. Our finding that α7nAChR activation by agonists stalls lesional progression is also consistent with the reported therapeutic potential of α7nAChR agonist [29], and is in agreement with our previous report that VNS impedes lesional progression [26]. Our finding that α7nAChR activation significantly retarded the development of endometriosis may explain as why nicotine or smoking has been identified as a protective factor for endometriosis [51] and adenomyosis [52] since nicotine is a ligand for AChRs.

What is puzzling is that treatment with MLA, an α7nAChR antagonist, did not seem to have much effect on lesions. It is possible that dosage that we used in this study was not optimized to affect lesions. It is also possible that there could be some redundant signaling pathways when α7nAChR is inhibited by an antagonist. For example, muscarinic AChR1 (m1AChR) in the forebrain has been reported to affect the neurons of the Medullary Visceral Zone (MVZ), which is the core of CAIP, to regulate systemic inflammation and immunity [53]. If endometriosis results in reduced vagal tone, it is possible that both α7nAChR and M1AChR are suppressed. If this is the case, then further suppression of α7nAChR may not be able to change much but its activation may be enough to activate the CAIP to exert desired therapeutic effects.

More remarkably, it is well-documented that women with endometriosis often exhibit anxiety, depression and insomnia [54–57]. These psychological co-morbidities themselves could activate the hypothalamic–pituitary–adrenal/sympatho-adreno-medullary (HPA/SMA) axes, and accelerate the development of endometriosis through adrenaline receptor β2 [58, 59]. This, in turn, is likely to cause more pain, anxiety and depression, forming a vicious cycle [60]. Yet anxiety, depression, and insomnia are reported to be associated with reduced vagal tone as well, which further justifies for VNS therapy [61–65]. Since activation of the CAIP is largely through the activation of α7nAChR [18, 19], targeting α7nAChR for therapeutic purpose may achieve the goal of not only regressing endometriotic lesions but also improving the overall wellbeing of the patients with endometriosis.

Our study has several strengths. First, by evaluating α7nAChR immunoreactivity in both OE and DE lesions, along with the extent of lesional fibrosis, we demonstrated that differential α7nAChR staining in different subtypes of endometriosis, which are known to have substantial histological differences [50, 66]. Second, through the use of in vitro and in vivo experimentations, we provided several, interlocking pieces of evidence for the therapeutic potentials of α7nAChR activation.

Our study also has some notable limitations. First, in mouse Experiment 1, we did not specifically quantitate the staining of either α7nAChR or m1AChR. However, PNU is known to be a specific agonist for α7nAChR [38, 67], while MLA is known to be a specific antagonist [39]. Yet quantification of m1AChR staining, perhaps in the MVZ may provide us with more information regarding the suppressed CAIP in mouse with induced endometriosis. Second, while we have demonstrated the effect of α7nAChR activation on a few major molecular events, such as EMT, FMT, SMM and fibrogenesis, involved in lesional progression, it is possible that there could be other pathways in retarding the progression. For example, thymic stromal lymphopoietin (TSLP) has been reported to be involved in the development of endometriosis [68–70], but α7nAChR has been shown to inhibit TSLP [71]. Future investigations are warranted to illuminate this issue.

In summary, we have shown in this study reduced α7nAChR in endometriotic lesions, especially in DE lesions. Through the impedance of EMT, FMT, SMM and fibrogenesis, pharmacological activation of α7nAChR decelerates the lesional progression in mouse and demonstrates its therapeutic potentials in mice with induced DE. Thus, α7nAChR can be viewed as a potential target for therapeutic invention.

## Supplementary Information

**Additional file 1: SupplementaryFigure S1. **Positive andnegative controls for immunohistochemistry. For positive controls, human breastcancer tissues were used for E-cadherin, mouse liver tissues for α-SMA, humanadenomyotic tissue samples for desmin and SM-MHC, and mouse lung tissues for α7nAChR.For negative controls, mouse endometriotic lesions were used. Magnification: 400×;Scale bar: 50 μm. **Supplementary Figure S2**. Cysticappearance of endometriotic lesions in mouse experiment 1. Lesions seen fromExperiment 2 are similar. **Supplementary** **Figure S3**. The number ofendometriotic lesions per mouse was evaluated from the Control, PNU-282987 andMLA groups in mouse experiment 1. Symbols forstatistical significance levels: NS: *p*>0.05; *: *p*<0.05;**: *p*<0.01. **SupplementaryFigure S4**. H&E staining from endometriotic lesions. Magnification = 200×, scale bar=100 μm. **Supplementary** **Figure S5**. The number of endometrioticlesions per mouse was evaluated from the Control and PNU-282987 groups in mouse experiment 2. NS: *p*>0.05. 

## Data Availability

The de-identified supporting data are available from the senior author upon written and reasonable request.
