# Supplementary material for: Activation of α7 nicotinic acetylcholine receptor retards the development of endometriosis
Source: Reprod Biol Endocrinol. 2022 Jun 4;20:85. doi: 10.1186/s12958-022-00955-w (PMC9166516; doi:10.1186/s12958-022-00955-w)
Supplement: Supplementary file 1 — Additional file 1: SupplementaryFigure S1. Positive andnegative controls for immunohistochemistry. For positive controls, human breastcancer tissues were used for E-cadherin, mouse liver tissues for α-SMA, humanadenomyotic tissue samples for desmin and SM-MHC, and mouse lung tissues for α7nAChR.For negative controls, mouse endometriotic lesions were used. Magnification: 400×;Scale bar: 50 μm. Supplementary Figure S2. Cysticappearance of endometriotic lesions in mouse experiment 1. Lesions seen fromExperiment 2 are similar. Supplementary Figure S3. The number ofendometriotic lesions per mouse was evaluated from the Control, PNU-282987 andMLA groups in mouse experiment 1. Symbols forstatistical significance levels: NS: p>0.05; *: p<0.05;**: p<0.01. SupplementaryFigure S4. H&E staining from endometriotic lesions. Magnification = 200×, scale bar=100 μm. Supplementary Figure S5. The number of endometrioticlesions per mouse was evaluated from the Control and PNU-282987 groups in mouse experiment 2. NS: p>0.05. [file 12958_2022_955_MOESM1_ESM.docx]

**Activation of α7 Nicotinic Acetylcholine Receptor**

**Retards the Development of Endometriosis**

**Meihua Hao, Xishi Liu, and Sun-Wei Guo.**

**Supplementary Information**

**
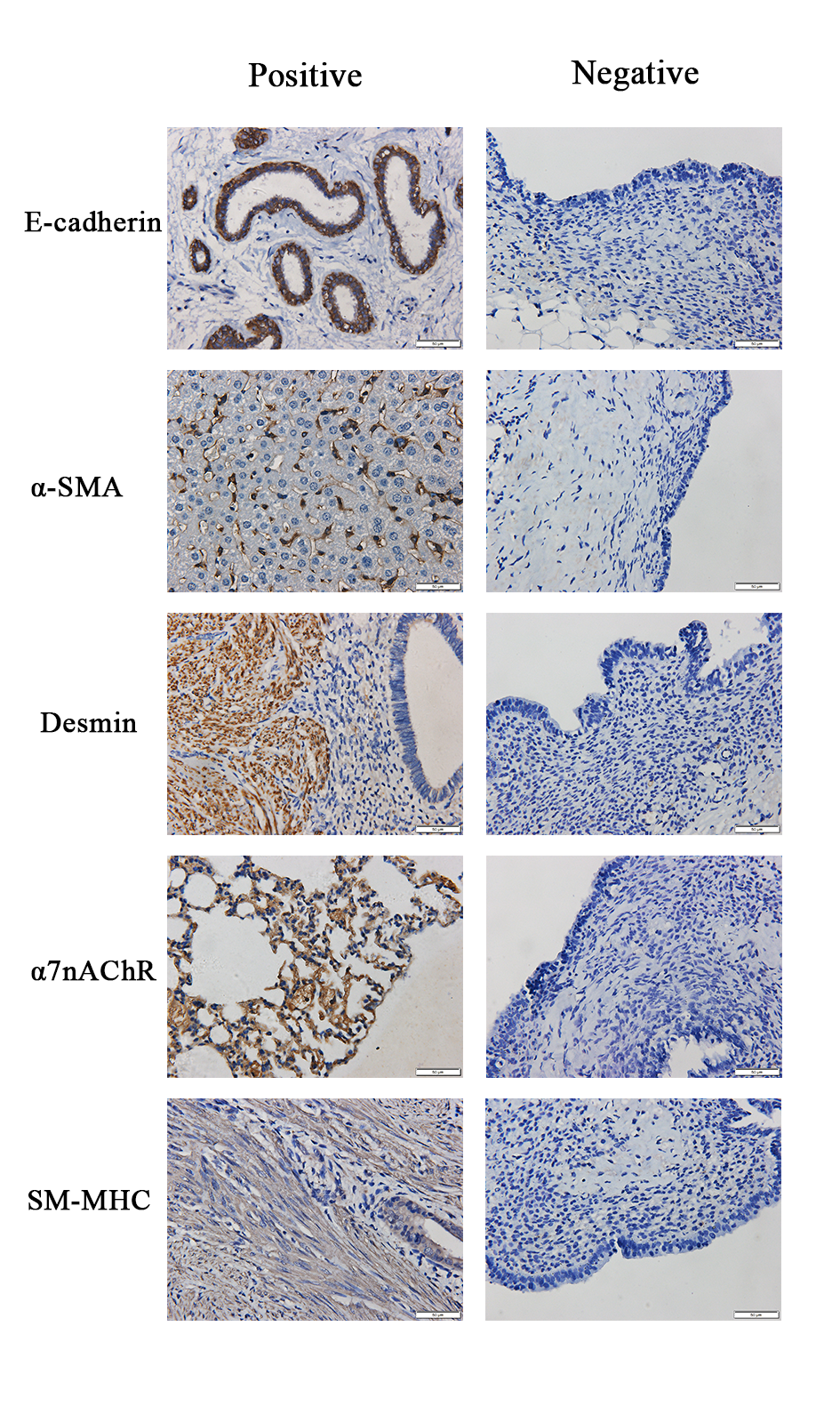
**

**Supplementary Figure S1**. Positive and negative controls for immunohistochemistry. For positive controls, human breast cancer tissues were used for E-cadherin, mouse liver tissues for α-SMA, human adenomyotic tissue samples for desmin and SM-MHC, and mouse lung tissues for α7nAChR. For negative controls, mouse endometriotic lesions were used. Magnification: 400×; Scale bar: 50 μm.


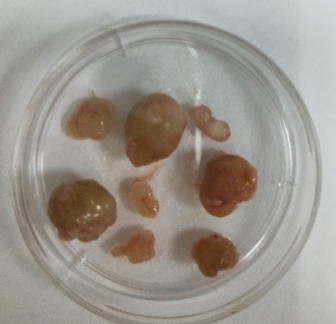


**Supplementary Figure S2**. Cystic appearance of endometriotic lesions in mouse experiment 1. Lesions seen from Experiment 2 are similar.


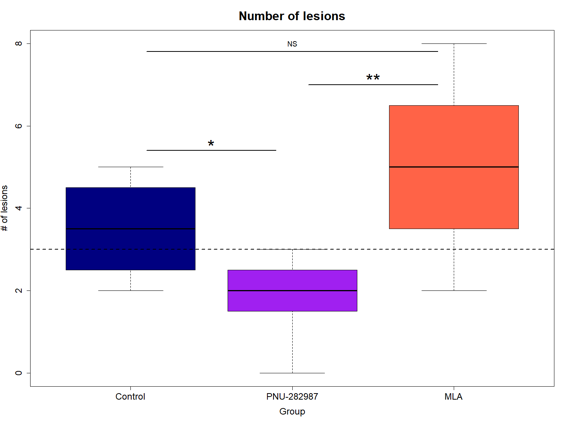


**Supplementary** **Figure S3**. The number of endometriotic lesions per mouse was evaluated from the Control, PNU-282987 and MLA groups in mouse experiment 1. Symbols for statistical significance levels: NS: p＞0.05; *: p<0.05; **: p<0.01.


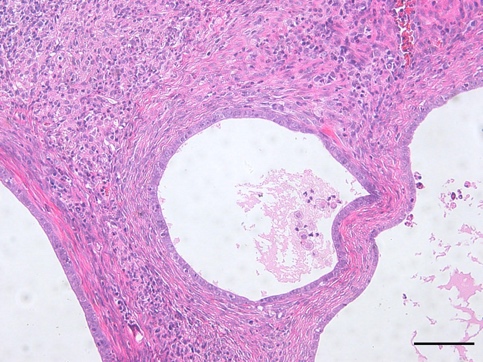


**Supplementary Figure S4**. H&E staining from endometriotic lesions. Magnification = 200×, scale bar= 100 μm.


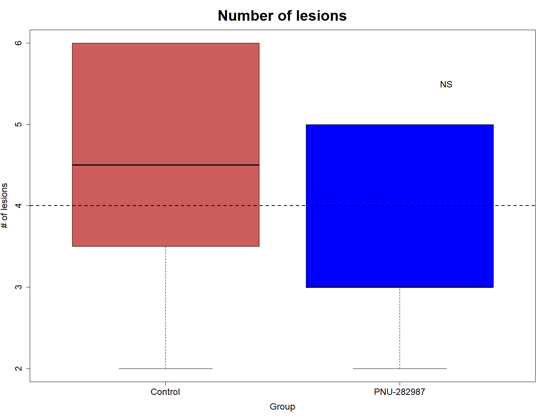


**Supplementary** **Figure S5**. The number of endometriotic lesions per mouse was evaluated from the Control and PNU-282987 groups in mouse experiment 2. NS: p＞0.05.
